# Bone Morphogenetic Protein-9 Enhances Osteogenic Differentiation of Human Periodontal Ligament Stem Cells via the JNK Pathway

**DOI:** 10.1371/journal.pone.0169123

**Published:** 2017-01-04

**Authors:** Pei Wang, Yanen Wang, Weizhong Tang, Xingxing Wang, Yanan Pang, Su Yang, Yibo Wei, Haochen Gao, Dalin Wang, Zhizhong Cao

**Affiliations:** 1 Department of Stomatology, Changhai Hospital, Second Military Medical University, Shanghai, China; 2 Clinical research center, Changhai Hospital, Second Military Medical University, Shanghai, China; University of Sheffield, UNITED KINGDOM

## Abstract

Bone morphogenetic protein-9 (BMP9) shows great osteoinductive potential in bone regeneration. Periodontal ligament stem cells (PDLSCs) with multi-differentiation capability and low immunogenicity are increasingly used as seed cells for periodontal regenerative therapies. In the present study, we investigated the potent osteogenic activity of BMP9 on human PDLSCs (hPDLSCs), in which the c-Jun N-terminal kinase (JNK) pathway is possibly involved. Our results showed that JNK inhibition by the specific inhibitor SP600125 or adenovirus expressing small interfering RNA (siRNA) targeting JNK (AdR-si-JNK) significantly decreased BMP9-induced gene and protein expression of early and late osteogenic markers, such as runt-related transcription factor 2 (Runx2), alkaline phosphatase (ALP), osteopontin (OPN), and osteocalcin (OCN), in hPDLSCs. We also confirmed the in-vivo positive effect of JNKs on ectopic bone formation induced by hPDLSCs injected into the musculature of athymic nude mice and BMP9 ex vivo gene delivery. For the cellular mechanism, we found that BMP9 activated the phosphorylation of JNKs and Smad2/3, and that JNKs may engage in cross-talk with the Smad2/3 pathway in BMP9-mediated osteogenesis.

## Introduction

Appropriate osteogenesis-regulating growth factors have been delivered to stem- cell-based tissues engineered to modulate the migration, proliferation and differentiation of progenitor cells for bone neogenesis. For stem-cell-based periodontal regeneration, periodontal ligament stem cells (PDLSCs) residing in the periodontium outperform bone marrow stromal cells and periosteal cells in formation of cementum, periodontal ligament (PDL)-like structures, and new bone [1,2]. Bone morphogenetic proteins (BMPs), which belong to the transforming growth factor β (TGF-β) superfamily, show great potential for promoting cell aggregation at the osteogenic site and inducing differentiation into osteoblasts [3,4]. BMP9, also known as the growth and differentiation factor 2 (GDF-2), was recently determined to be capable of inducing osteogenic differentiation and bone formation both in vitro and in vivo [5,6]. Among 14 types of BMPs investigated in vivo, BMP9 was the most potent inducer of osteogenic differentiation, although BMP-2, -6, and -7 could induce bone tissue-associated gene mRNA expression and mineralization of human PDLSCs (hPDLSCs) [7]. So far, few studies have investigated the osteogenic effect of BMP9 on hPDLSCs and its role in periodontal bone regeneration. Therefore, we investigated whether delivery of an adenovirus-mediated BMP9 gene would promote the proliferation and osteogenic differentiation of hPDLSCs.

Although BMP9 is among the most osteogenic BMPs, it remains the least characterized BMP and little is known about the precise cellular signal transduction mechanism involved in BMP9-induced osteogenesis. In addition to the congenital Smad signaling pathway, the mitogen-activated protein kinase (MAPK) pathway probably plays a vital role in BMP-induced osteogenesis of mesenchymal stem cells (MSCs) [8,9]. As reported, p38 and extracellular signal-regulated kinases (Erk1/2), subfamilies of MAPKs, have opposite regulatory effects on BMP9-induced osteogenic differentiation of PDLSCs [6,10]. The activation of JNK, another important MAPKs member, has a stimulatory effect on BMP9-induced osteogenic differentiation of MSCs [11]. However, there have been no reports about the involvement and concrete role of JNKs in BMP9-mediated osteogenesis of hPDLSCs. In this study, we found that JNK inhibition by inhibitor SP600125 or siRNA suppressed the gene and protein expression of Runx2, ALP, OPN, and OCN in BMP9-induced osteogenic differentiation, suggesting that JNKs play an important role in BMP9-induced osteogenic differentiation of hPDLSCs. To our knowledge, there are no experimental reports addressing the use of hPDLSCs with BMP9 as an ex vivo gene therapy to induce ossification in vivo. Thus, we innovatively investigated whether BMP9 gene therapy with hPDLSCs can induce ectopic bone formation in the thigh musculature of athymic nude mice, as well as the role of JNKs in this process. Our radiography and histological results showed that JNK inhibition by siRNA dramatically decreased BMP9-induced osteoblast differentiation and maturation in vivo. Taken together, these results suggest that JNK has a positive effect on BMP9-induced osteogenic differentiation of hPDLSCs both in vitro and in vivo. JNK, a member of the TGF-β family, has been demonstrated to be rapidly activated by TGF-β in a Smad-independent pathway, and JNKs phosphorylate Smad3 outside of its -SSXS motif, which further promotes its activation by the TGF-β receptor complex and its nuclear accumulation for the activation of its transcriptional partner [12]. For the cellular mechanism, we also found that JNKs interact with the Smad2/3 pathway during osteogenic differentiation stimulated by BMP9.

## Materials and Methods

### Ethics Statement

This study protocol was approved by the Ethical Review Committee for Research on Human Subjects of the Second Military Medical University. In this study, all isolated human premolars for orthodontic purpose were collected with written informed consent from the children’s parents/guardians prior to study participation. This study is in strict accordance with approved guidelines set by the National Institute of Health Office of Human Subjects Research, regulations on the management of medical waste, and the principles expressed in the Declaration of Helsinki.

### Sample Collection and Cell Culture

PDL tissues were collected from normal premolars (n = 12) of 6 patients (mean age 15 years) for orthodontic purposes at the Department of Stomatology in Changhai Hospital. The experimental protocol was approved by the Ethics Committee of the Second Military Medical University. PDL tissues were removed from the middle third of the root surfaces. The obtained tissue fragments were rinsed two times with -phosphate buffered saline (PBS) and then placed evenly into 6-cm-diameter culture dishes containing a growth medium (GM). The GM was Dulbecco's modified Eagle's medium with 4.5 g/L glucose, L-glutamine & sodium pyruvate (DMEM, Corning, Manassas, VA) supplemented with 10% fetal bovine serum (FBS, Gibco, Grand Island, NE), 200 mM L-glutamine, 100 U/mL penicillin and 100 mg/mL streptomycin. The disperse tissues were gently covered with coverslips to ensure better adherence of the tissues to the dishes. Finally, the dishes were incubated at 37°C with 5% CO_2_, and the medium was refreshed every three days until the cells successfully grew from the tissue blocks. The cells, after reaching 80%-90% confluence, were subcultured in trypsin/ethylene diamine tetraacetic acid (EDTA).

### Immunomagnetic Separation and Flow Cytometry Analysis

The immunomagnetic isolation method was reported previously. Briefly, 1×10^7^ third-passage hPDLCs were incubated in the dark at 4°C for 20 min with 60 μL of a buffer containing 0.5% bovine serum albumin (BSA), 2 mM EDTA, 20 μL of CD146-conjugated magnetic beads and 20 μL of FcR Blocking Reagent. The bead-positive cells were separated using a CD146 microbead kit, LS Columns, and a MACS separator, according to the manufacturer’s instructions (Miltenyi Biotec, Bergisch Gladbach, Germany). After that, cells that were positive or negative for CD146 antigen were subjected to flow cytometry to determine the stem cell markers STRO-1 and CD146. Approximately 5×10^5^ CD146^+^ PDLSCs were centrifuged at 400×g for 10 min, re-suspended in a blocking buffer (PBS containing 5% FBS) on ice for 20 min and stained with fluorescein isothiocyanate (FITC)-conjugated monoclonal antibodies against human STRO-1 (Abcam, Cambridge, UK) and allophycocyanin-conjugated monoclonal antibodies against human CD146 (Miltenyi Biotec) on ice in the dark for 30 min. STRO-1^+^/CD146^+^ cells were detected by a flow cytometer vantage cell sorter (Miltenyi Biotec), and data were analyzed on FlowJo 7.6.1 software (FlowJo, USA)

### Multi-Differentiation Potential of hPDLSCs

To investigate the multipotency of PDLSCs, the osteogenic and adipogenic differentiation potential was determined. For osteogenic differentiation, the isolated CD146^+^ PDLCs were seeded onto 6-well plates at a density of 1×10^5^ cells/well in GM. The cells, after reaching 80% confluence, were incubated in an osteogenic medium (OM; 100 nM dexamethasone, 50 mg/ml ascorbic acid, and 5 mM β-glycerophosphate; Sigma-Aldrich, St. Louis, MO) for 21 days, followed by cell assay for calcium deposits using Alizarin Red S staining (Sigma-Aldrich, St. Louis, MO). For adipogenic differentiation, the cells were cultured first in an adipogenic induction medium (basic medium supplemented with 1 μM dexamethasone, 10 μM insulin, 0.5 mM 1-methyl-3-isobutylxanthine, and 200 μM indomethacin) for 3 days and then transferred to a maintenance medium (basic medium supplemented only with 10 μM insulin) for 3 days. After culture for 21 days, the cells were fixed with 4% paraformaldehyde (PFA) and stained with Oil Red O (Sigma-Aldrich, St. Louis, MO).

### Immunofluorescence (IF) Staining

IF assays were used to determine the origin of these cells. Briefly, the cells were seeded in 6-well plates at a density of 1×10^6^ cells/well and cultured in a complete medium. When reaching 60%–70% confluence, the third-passage PDLCs were treated with 4% PFA for 30 min, permeabilized with 0.2% Triton X-100, and then washed with PBS. After that, the cells were incubated with mouse primary antibodies against human vimentin and human cytokeratin (both Abcam, 1:1000) overnight at 4°C for 30 min, and then with fluorescence-conjugated goat anti-mouse IgG secondary antibodies. Nuclei were stained with 4',6-diamidino-2-phenylindole (Beyotime, Shanghai, China). Fluorescence signals were recorded under an inverted fluorescence microscope (Olympus, Tokyo, Japan).

### Construction of Recombinant Adenoviruses

Replication-deficient recombinant adenoviruses expressing BMP9 (Ad-BMP9) and helper green -fluorescent protein (Ad-GFP), or expressing siRNA (Ad-siRNA) targeting JNK1/2 (AdR-si-JNK) (5’-GCCCAGUAAUAUAGUAGUATT-3’) and helper mCherry -fluorescent protein (Ad-mCherry) were purchased from Hanbio (Shanghai, China). A pHBAd-MCMV-GFP or pHBAd-MCMV-mCherry overexpression vector was constructed to mediate the delivery of target genes to cells. The adenovirus transfections were performed according to the manufacturer’s instructions.

### GFP or mCherry Expression and Infectivity Test

The isolated third-passage hPDLSCs were seeded onto 96-well plates at a density of 5×10^3^ cells/well. Once the cells reached 100% confluence or post-confluence, they were infected with Ad-GFP or Ad-mCherry at different viral concentrations (10, 20, 30, 60, multiplicities of infection (MOI)), according to the manufacturer’s instructions. The appropriate MOI of the transfected adenoviruses was determined by observing the cell microscopic fluorescence area for two consecutive days. The expression of GFP or mCherry protein was observed under an inverted fluorescence microscope 48h post- infection.

### ALP Assay

To detect the effect of BMP9 on the ALP activity of hPDLSCs, and in which the detail effect of JNKs pathway, we examined ALP activity in cell lysates of different treatment groups quantitatively at the indicated time points using an ALP assay kit (Beyotime, Shanghai, China). Firstly, to determine the effect of BMP9 on the ALP protein expression of cells, the CD146^+^ hPDLSCs were cultured in 96-well cell culture plate at a density of 5×10^3^ cells/well and transfected with Ad-BMP9 (treatment group) or Ad-GFP (control group) when reaching 80%-90% confluence. Next, we use JNKs inhibitor SP600125 or AdR-si-JNK to investigate the effect of JNKs pathway on the ALP activity of hPDLSCs stimulated by BMP9. hPDLSCs were seeded in 96-well plate and transfected with BMP9, followed by treated with 5, 10, or 15 μmol/L SP600125 (Selleck, Houston, TX) which were refreshed with new JNK inhibitor portions every three days, or infected with AdR-si-JNK after 48h of BMP9 transfection. At 4, 7, and 10 days after cultured, the cells were washed with PBS and incubated in the dark for 30 min. Finally, ALP activity in cell lysates was analyzed by measuring the optical density (OD) at 405 nm on a microplate reader (Varioskan Flash, Waltham, CT). Each condition was tested in sextuple, and the experiment was repeated three times.

### Quantitative Real-time Polymerase Chain Reaction (qRT-PCR)

We deteted the effect of BMP9 on osteogenic-related gene expression of Runx2, ALP, OPN, and OCN of hPDLSCs, and in which the possible involvement of JNKs pathway via qRT-PCR analysis. The isolated CD146^+^ PDLSCs were seeded onto 6-well plate at a density of 1×10^5^ cells/well. After reaching 80% confluence, the cells were infected with Ad-BMP9, followed by treatment with SP600125, or co-infected with AdR-si-JNK after 48h of BMP9 infection. Total RNA was extracted from cells with Trizol reagent (TaKaRa Bio, Kyoto, Japan) and then converted to cDNA by a PrimeScript^TM RT Master Mix (TaKaRa Bio, Kyoto, Japan). The target genes were amplified with an SYBR RT-PCR Kit (TaKaRa Bio, Kyoto, Japan) on LightCycler® 480Ⅱ(Roche, Basel, Swiss). The cycling program was as follows: 95°C for 5 s, 60°C for 15 s, 72°C for 15 s, for 45 cycles. All experiments were run in triplicate. Each gene cycle threshold (ct) was normalized based on the ct of β-actin examined simultaneously on the same plate, and then calculated by 2^-ΔΔCt^. The primer sequences for BMP9, Runx2, ALP, OPN, OCN, and β-actin were synthesized based on GeneBank, as follows:

1. β-actin: forward (5’-GCCAACACAGTGCTGTCT-3’), reverse (5’-AGGAGCAATGATCTTGATCTT-3’)
2. BMP9: forward(5’-AGAACGTGAAGGTGGATTTCC-3’), reverse (5’-CGCACAATGTTGGACGCTG-3’)
3. ALP: forward (5’-GTTGCCAAGCTGGGAAGAACAC-3’), reverse (5’-CCCACCCCGCTATTCCAAAC-3’)
4. Runx2: forward (5’-TAGATGGACCTCGGGAACC-3’), reverse (5’-GGGTGGTAGAGTGGATGGAC-3’)
5. OPN: forward (5’-GCCGAGGTGATAGTGTGGTT-3’), reverse (5’-CTGGACTGCTTGTGGCTGT-3’)
6. OCN: forward (5’-GGCAGCGAGGTAGTGAAGAG-3’), reverse (5’-CTGGAGAGGAGCAGAACTGG-3’) (Shengong, shanghai, China)

### Western Blotting Analysis

To investigate the effects of BMP9 on the protein expression of ALP, OPN, OCN, p-Smad2/3 and p-JNK, we incubated the BMP9-infected hPDLSCs in the presence or absence of different concentrations of SP600125 or co-infected with AdR-si-JNK. Cells were lysed in a radioimmunoprecipitation assay (RIPA) buffer (Beyotime, Shanghai, China). After centrifugation, proteins in the supernatant were collected and quantified using a bicinchoninic acid (BCA) protein assay kit (Beyotime, Shanghai, China). The total proteins were denatured by boiling for 5 min, resolved by 10% gradient SDS-PAGE and then transferred to PVDF membranes. After blocking with 5% BSA for 2 h, the membranes were incubated with 1:2000 primary antibodies against anti-ALP, anti-OPN, anti-OCN, anti-p-Smad2/3, anti-p-JNK1/2 (all from Abcam, Cambridge, UK) and anti-glyceraldehyde 3-phosphate dehydrogenase (GAPDH) as housekeeping gene (Beyotime, Shanghai, China) overnight, followed by the addition of HRP AffiniPure goat anti-rabbit IgG(H+L) (Abcam, Cambridge, UK) as the secondary antibody for 2 h. The blots were visualized by enhanced chemiluminescence (ECL) and autoradiography.

### Alizarin Red Staining

Third-passage hPDLSCs were seeded into a 6-well cell culture plate at a density of 5×10^4^ cells/well. When cells reached 100% confluence or post-confluence, hPDLSCs were transfected with Ad-BMP9 or co-transfected with Ad-BMP9 and Ad-siR-JNK for 24 h, and then, the GM was replaced with OM. Subsequently, BMP9-transfected hPDLSCs were treated with different concentrations of SP600125 (5, 10, or 15 μmol/L). After osteogenic induction for three weeks, the cells were fixed with 4% paraformaldehyde and extracellular matrix mineralization in the different treatment groups was evaluated using Alizarin Red staining (Cyagen Biosciences, Chicago, IL).

### Histological Analysis and Cone Beam Computer Tomography (CBCT) Analysis

This study was carried out in strict accordance with the recommendations in the Guide for the Care and Use of Laboratory Animals of the National Institutes of Health. The in vivo experimental protocols used in this study were approved by the Institutional Animal Care and Committee on Ethics of Biomedicine Research, Second Military Medical University. All surgery was performed under sodium pentobarbital anesthesia, and all efforts were made to minimize suffering.

Before implantation, hPDLSCs were infected with optimal concentrations of Ad-BMP9 (MOI 30–60) alone or co-infected with AdR-si-JNK (MOI 30–60). Twelve 4-week-old female athymic nude mice (BALB/c nu/nu) (4 mice per group) were anesthetized by intraperitoneal injection with pentobarbital (Nembutal 3.5 mg/100 g). Then, the mice received a percutaneous injection (a suspension of 1×10^6^ hPDLSCs or Ad-BMP9-transfected cells or Ad-BMP9 and AdR-si-JNK co-infected cells at 30 PFU/cell) into the thigh musculature to determine the role of the JNKs pathway in BMP9-induced osteogenesis of hPDLSCs. Two months after transplantation, the mice were euthanized with CO_2_, the heterotopic ossification implants were removed and examined radiologically and histologically by CBCT scans (i-CAT17-19, Hatfield, PA), and hematoxylin, and eosin (H&E) staining. Harvested implants were demineralized, fixed in 4% formalin overnight, paraffin-embedded, and then stained with hematoxylin and eosin (H&E). The in vivo implants were scanned by CBCT and the three-dimensional images were reconstructed using i-CATVision software. The mean bone mineral density (BMD) of the implant sites was measured by 3DVR software, and the results were recorded using Hounsfield units (HU).

### JNKs and Smad2/3 Pathway Crosstalk in BMP9-Mediated Osteogenesis

To investigate the relationship between JNKs and the Smad2/3 pathway in BMP9-induced osteogenesis of hPDLSCs, the effect of JNKs inhibition on the expression of p-Smad2/3 and total Smad2/3 in hPDLSCs stimulated by BMP9, was detected with a western blotting assay. First, BMP9-transfected hPDLSCs were treated with the JNK inhibitor SP600125 at various concentrations or co-infected with AdR-si-JNK for 48 h. Then, the western blotting assay which has been mentioned above was performed to detect the protein expression of p-Smad2/3 in the different treatment groups.

Next, we performed a co-immunoprecipitation (co-IP) assay to investigate heteromeric complex formation of endogenous phosphorylated JNK (p-JNK) and Smad2/3 in BMP9-transfected hPDLSCs. We first examined the input proteins Smad2/3 and p-JNK by a western blotting assay in the BMP9 or GFP transfection group and then identified the complex formations of both with co-IP analyses. For the co-IP assay, at 72 h after BMP9 or GFP transfection, the proteins in the cell lysates were collected and quantified using a BCA protein assay kit. After blocking with BSA, the blots were incubated with primary antibodies such as rabbit anti-Smad2/3 antibody (Abcam), and rabbit anti-IgG antibody (Abcam) as the control. Then, mouse anti-p-JNK1/2 antibody (Abcam) was added to detect the formation of protein Smad2/3 and p-JNK complexes. After washing, the immunoreactive proteins were visualized by ECL and autoradiography.

### Statistical Analysis

All data are expressed as the mean±standard deviation (SD). Statistical analyses were performed on SPSS 19.0 software. Differences between groups were examined via one-way ANOVA, and Tukey’s multiple comparisons test, with a significance level of p<0.05.

## Results

### Isolation and Characterization of hPDLSCs

**Fig 1:**
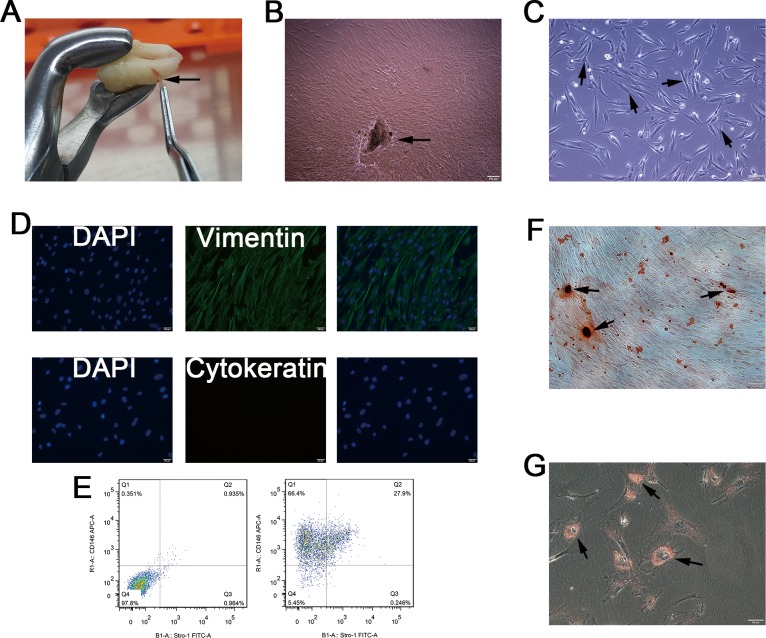
Isolation and characterization of human PDLSCs. (**A**) We isolated PDLCs from PDL tissues (arrow), which are attached to the surface of the roots. (**B**) Cell clusters, derived from tissue blocks (arrow) showed radiating or whirlpool-like morphology. (**C**) Isolated CD146^+^ PDLCs were small, round, fusiform, and triangular (arrow). The cells had abundant cytoplasm and large nuclei, and several binucleate cells were observed (arrow). (**D**) Immunofluorescence staining showed that isolated CD146^+^ PDLCs stained positive for vimentin, and negative for cytokeratin. (**E**) Flow cytometry analysis showed that the percentage of cells expressing the stem cell surface markers STRO-1 and CD146 was 27.9%, after CD146 immunomagnetic isolation. (**F**) Alizarin Red staining showed the formation of some mineralized nodules in cultured PDLSCs after osteogenic induction for 3 weeks. (**G**) PDLSCs formed Oil Red O-positive lipid droplets after 3 weeks of induction in the adipogenic medium.

PDLSCs were isolated from a human PDL cell population using CD146-conjugated immunomagnetic microbeads (Fig 1A). The isolated cells formed colonies, which showed radiating or whirlpool-like morphology (Fig 1B). Most cells exhibited a fibroblastic spindle, and a small round or triangular shape, and most cells had abundant cytoplasm and a large nucleus (Fig 1C). IF staining showed that the isolated PDLCs primarily expressed vimentin rather than cytokeratin, which suggests their mesodermal origin (Fig 1D). The stem cell STRO-1^+^/CD146^+^ immunophenotype which are expressed by hPDLSCs, was determined by flow cytometry. The mean percentage of the STRO-1^+^/CD146^+^ cells in PDLCs populations after immunomagnetic isolation was 27.9% (Fig 1E). In addition, Alizarin red S staining after three weeks of osteo-induction showed that some mineralized nodules were formed in the isolated PDLSCs (Fig 1F). Similarly, Oil Red O-positive lipid droplets were observed in PDLSCs after three weeks of adipogenic induction (Fig 1G). These results confirm the multi-differentiation potential of the isolated PDLSCs, which could then be used for subsequent experiments.

### Ad-GFP Infection Efficiency and Overexpression of BMP9 in hPDLSCs

**Fig 2:**
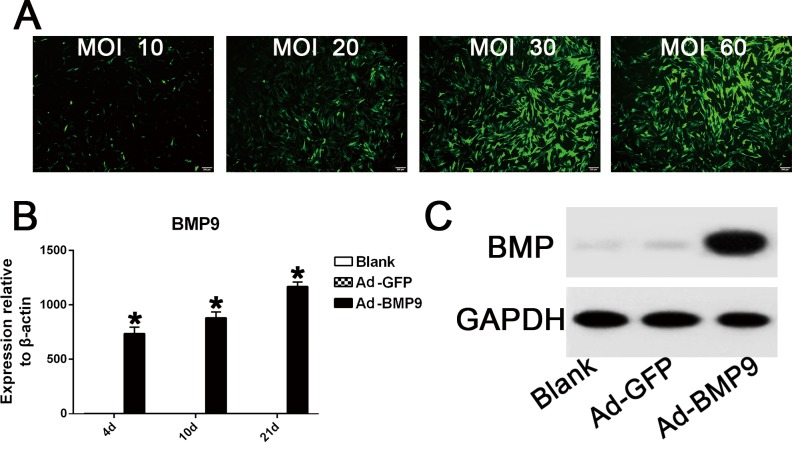
Helper GFP infection efficiency and overexpression of BMP9 in hPDLSCs following BMP9 transfection. (**A**) The fluorescence area of GFP-positive cells increased gradually when the MOI value ranged from 10 to 30, while the GFP infective efficiency remained similar between the 30 MOI group and the 60 MOI group. Magnification, 100×. (**B**) qRT-PCR results showed that the BMP9 transfection group had 700–1200 fold higher gene overexpression of BMP9 compared to the control GFP group. “*”, p<0.01 (vs. control groups). (**C**) Western blot assay suggested that in the Ad-BMP9 transfection group, BMP9 protein expression was significantly promoted until day 21.

Observation of the areas of fluorescence, using a fluorescence microscope, showed that the number of GFP-positive cells increased gradually when the adenoviral concentrations ranged from 10 to 30 MOI (Fig 2A). However, the fluorescence area did not increase in the range from 30 to 60 MOI (MOI 30–60 PFU/cell), in which the transduction efficiency was approximately 70%-80% (Fig 2A). Moreover, cell morphology and viability were found normal in either MOI 30 or 60 groups. Therefore, the optimal concentration for adenovirus infection was determined to be 30–60 MOI. qRT-PCR and western blot results showed that the Ad-BMP9-transfected cells had stable and high BMP9 gene expression throughout the osteogenic differentiation process of hPDLSCs (Fig 2B and 2C).

### BMP9 Promoted Osteogenic Differentiation of hPDLSCs

**Fig 3:**
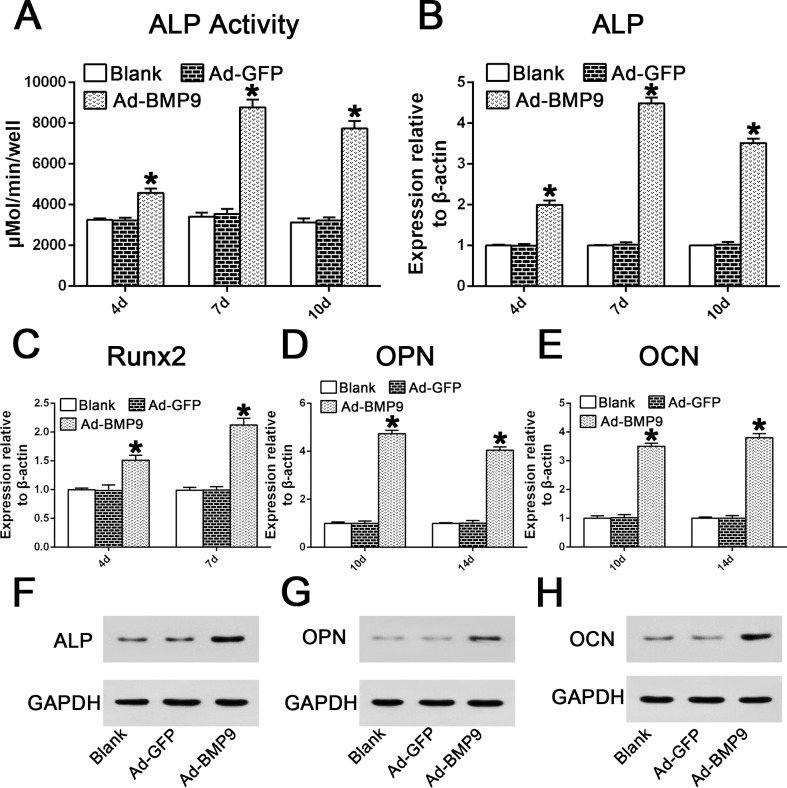
Effects of BMP9 on the mRNA gene and protein expression of osteogenic markers in hPDLSCs. (**A-B**) Data are presented as the mean±SD and the relative expression level of each gene was normalized to β-actin. The expression of ALP was measured via a quantitative ALP activity kit and qRT-PCR analysis at days 4, 7, and 10 in the early osteogenic phase of hPDLSCs. “*”, p<0.01 (vs. control groups). (**C**) qRT-PCR analysis of BMP9-induced Runx2 mRNA expression on days 4 and 7. “*”, p<0.01 (vs. control groups). (**D-E**) qRT-PCR analysis of BMP9-induced OPN and OCN gene expression on days 10 and 14. “*”, p<0.01 (vs. control groups). (**F**) Western blotting suggested that BMP9 significantly enhanced ALP protein expression on day 7. (**G**) Western blotting assay showed that BMP9 stimulated OPN protein expression on day 10. (**H**) Western blotting assay showed that BMP9 stimulated OCN protein expression on day 14.

We investigated the effect of BMP9 on ALP activity, a well-established early osteogenic differentiation marker, in PDLSCs at the indicated time points. As expected, the ALP activity in the BMP9-transduced hPDLSCs at day 4, day 7, and day 10 increased significantly by 1.4-, 2.57-, and 2.47-fold, respectively, compared with the control GFP group (Fig 3A; p<0.01). Similarly, BMP9 also significantly up-regulated the gene expression of ALP by 1.99-, 4.4- and 3.52-fold on days 4, 7, and 10, repectively, compared to the control group (Fig 3B; p<0.01). The mRNA expression levels of Runx2, another master gene regulating early osteogenic differentiation, after BMP9 stimulation at days 4 and 7, were 1.51- and 2.12-fold higher compared with the GFP group (Fig 3C; p<0.01). In addition, BMP9 also up-regulated intermediate and late osteogenic markers such as OPN (by 4.73- and 4.05-fold) (Fig 3D; p<0.01) and OCN (by 3.5- and 3.8-fold) (Fig 3E; p<0.01) at days 10 and 14. Furthermore, western blot results also showed that the protein expression of ALP, OPN, and OCN was stimulated by the overexpression of BMP9 at the indicated time points (Fig 3F–3H). Thus, these results confirm the osteo-inductive potential of BMP9 in early and late osteogenic differentiation of hPDLSCs.

### Inhibition of JNKs by Inhibitor SP600125 Decreased BMP9-Induced Osteogenic Differentiation of hPDLSCs

**Fig 4:**
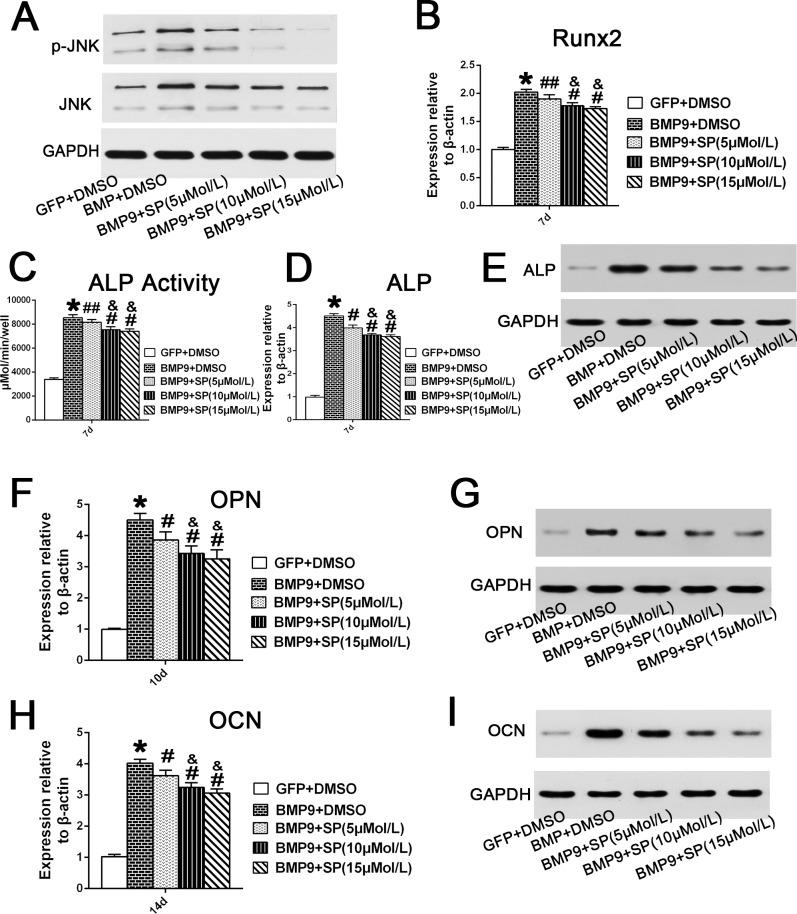
Inhibition of JNKs suppresses BMP9-induced osteogenic differentiation of hPDLSCs. (**A**) Total JNK and phosphorylated JNK in Ad-BMP9-transfected hPDLSCs in the presence of SP600125 (5, 10, or 15 μM) were analysed by western blotting at day 4. (**B**) The Runx2 mRNA expression in BMP9-transfected cells was assessed by qRT-PCR assay after treatment with SP600125 (5, 10, or 15 μM) for 7 days. ‘‘*”, p<0.01 (vs. control group); ‘‘#”, p<0.01 (vs. BMP9 group); ‘‘##”, p<0.05 (vs. BMP9 group); “&”, p<0.01(vs. SP 5uM group); There was no significant difference between the SP 10 μM group and SP 15 μM group. (**C**) and (**D**) and (**E**) ALP activity in Ad-BMP9-transfected hPDLSCs in the presence of with SP600125 (5, 10, or 15 μM) was assessed by quantitative assay, qRT-PCR and western blotting assay at day 7. ‘‘*”, p<0.01 (BMP9 group vs. GFP control group); ‘‘#”, p<0.01 (vs. BMP9); ‘‘##”, p<0.05 (vs. BMP9 group); “&”, p<0.01(vs. SP 5 μM group). There was no significant difference between the SP 10 μM group and SP 15 μM group. (**F**) and (**G**) The mRNA expression and protein expression of OPN in BMP9-transfected cells after treatment with SP600125 (5, 10, or 15 μM) for 10 days, were analyzed by qRT-PCR and western blotting. ‘‘*”, p<0.01 (vs. control group); ‘‘#”, p<0.01 (vs. BMP9 group); “&”, p<0.01(vs. SP 5 μM group); There is no significance between SP 10 μM group and SP 15 μM group. (**H**) and (**I**) The mRNA expression and protein expression of OCN in hPDLSCs infected with BMP9 in the presence of SP600125 (5, 10, or 15 μM) for 14 days were analyzed by qRT-PCR and western blotting. ‘‘*”, p<0.01 (vs. control group); ‘‘#”, p<0.01 (vs. BMP9 group); “&”, p<0.01(vs. SP 5 μM group); There was no significant difference between the SP 10 μM group and the SP 15 μM group.

SP600125, a specific JNKs/MAPK inhibitor, was used to determine the role of JNKs in BMP9-induced osteogenic differentiation of hPDLSCs. The inhibitory effect of SP600125 on JNK phosphorylation 4 days after BMP9 transfection was assessed through western blotting. The results showed that BMP9 stimulation increased JNKs phosphorylation, while the total amount of JNK was unaffected, and SP600125 decreased the amount of p-JNK in hPDLSCs in a dose-dependent manner (Fig 4A). We detected Runx2 gene expression in BMP9-transfected hPDLSCs after treating with different concentrations of SP600125 (5, 10 and 15 μM). The qRT-PCR results showed that SP600125 suppressed Runx2 mRNA expression in hPDLSCs stimulated by BMP9 in a dose-dependent manner at day 7 (Fig 4B; p<0.05). Moreover, we further detected ALP protein and mRNA transcription levels at day 7 by quantitative assay, qRT-PCR and western analysis. Our results revealed that the mRNA and protein expression of ALP in hPDLSCs was dose-dependently reduced by the JNK inhibitor (Fig 4C–4E; p<0.01). Similarly, qRT-PCR and western blot results also demonstrated that SP600125 had an inhibitory effect on OPN gene and protein expression at day 10 (Fig 4F and 4G; p<0.01). Two weeks post osteo-induction, our qRT-PCR and western blotting analysis suggested that SP600125 treatment significantly decreased the BMP9-induced OCN gene and protein expression at day 14 (Fig 4H and 4I; p<0.01). These results strongly suggest that JNKs play a positive regulatory role in BMP9-induced osteogenesis of hPDLSCs.

### JNK Gene Silencing Negatively Regulated BMP9-Induced Osteogenic Differentiation of hPDLSCs

**Fig 5:**
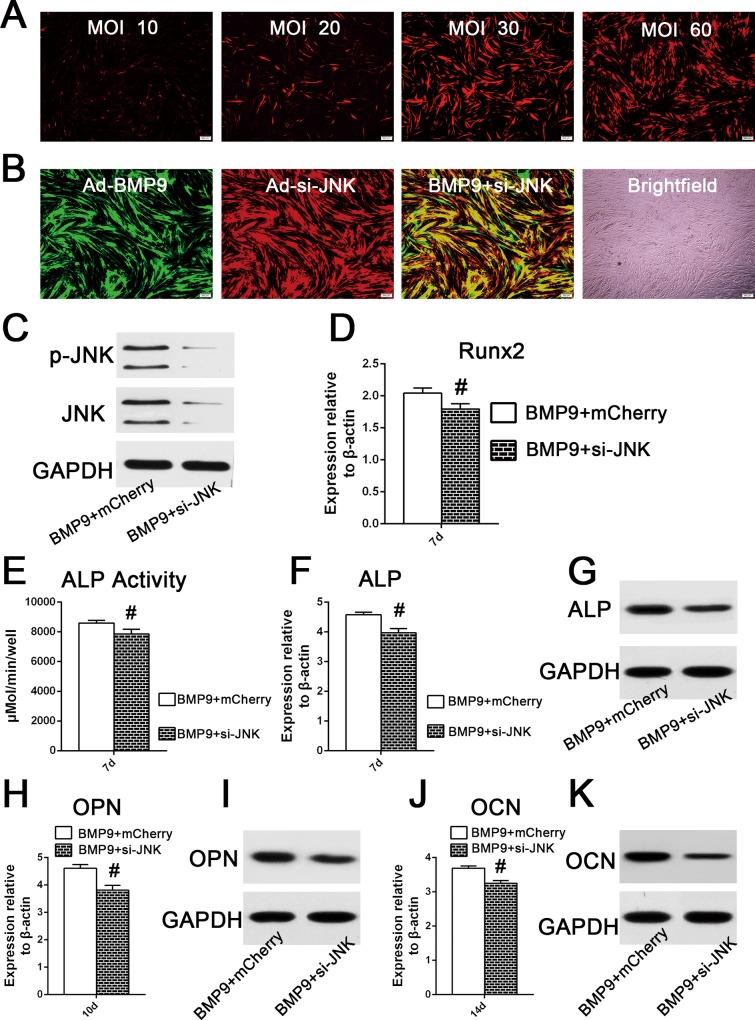
JNKs knockdown decreased BMP9-stimulated osteogenic differentiation of hPDLSCs. (**A**) The helper mCherry infective efficiency was determined by the observation of the area of fluorescence after hPDLSCs were transfected with different adenoviral concentrations (10, 20, 30, or 60 MOI) for 2 days. (**B**) When hPDLSCs were co-infected with Ad-GFP (MOI 30–60) and Ad-mCherry (MOI 30–60), the co-infective efficiency was approximately 80%, according to observation of the area of fluorescence 72h post co-infection. (**C**) Western blot analysis showed that si-JNK significantly decreased the phosphorylation of JNK1/2 in hPDLSCs stimulated by BMP9, after 72h of co-infection. (**D**) Effect of JNK1/2 gene silencing on the mRNA expression of Runx2 in hPDLSCs stimulated by BMP9, determined by qRT-PCR analysis at day 7. ‘‘#”, p<0.01 (vs. BMP9 group). (**E**) and (**F**) and (**G**) Effect of JNK1/2 knock down on the gene and mRNA expression of ALP in hPDLSCs stimulated by BMP9 by a quantitative ALP activity kit, qRT-PCR assay, and western blotting. ‘‘#”, p<0.01 (vs. BMP9 group). (**H**) and (**I**) Effect of JNK1/2 gene silence on the mRNA and protein expression of OPN in hPDLSCs stimulated by BMP9, was determined by qRT-PCR and western analysis at day 10. ‘‘#”, p<0.01 (vs. BMP9 group). (**J**) and (**K**) Effect of JNK1/2 gene silencing on the mRNA and protein expression of OCN in hPDLSCs stimulated by BMP9, was determined by qRT-PCR and western analysis at day 14. ‘‘#”, p<0.01 (vs. BMP9 group).

To exclude the possibility that the osteogenic differentiation inhibition by SP600125 was the result of a nonspecific drug effect, we next examined the effect of Ad-siRNA-mediated JNK1/2 knockdown on BMP9-induced osteoblastic differentiation of hPDLSCs. Similar to the GFP infective efficiency test, the optimal transfection concentration of adenoviruses expressing mCherry was determined to be in the MOI range of 30–60 (Fig 5A). Observation of the fluorescence area showed that the transduction efficiency was approximately 80% when hPDLSCs were co-transfected with Ad-BMP9 and Ad-mCherry (MOI 30–60) (Fig 5B). Western blotting showed that AdR-si-JNK significantly inhibited the phosphorylation of JNKs (Fig 5C). The effect of JNK gene silencing on the BMP9-stimulated osteogenic differentiation of hPDLSCs was also investigated. We found that JNK1/2 knockdown reduced Runx2 gene expression by 0.88-fold at day 7 (Fig 5D; p<0.01). Furthermore, the results of a quantitative assay, qRT-PCR and western blot analysis showed that JNK1/2 gene silencing significantly inhibited the osteogenesis-related gene and protein expression of ALP at day 7 (Fig 5E–5G; p<0.01). The gene and protein expression of intermediate and late markers regulating osteogenic differentiation, such as OPN and OCN, stimulated by BMP9 were also inhibited by si-JNK treatment (Fig 5H–5K; p<0.01). Taken together, these results indicate that JNKs are likely to play a positive regulatory role in BMP9-induced osteogenic differentiation of hPDLSCs.

### Inhibition of JNKs Reduced the Extracellular Mineralization of hPDLSCs Stimulated by BMP9

**Fig 6:**
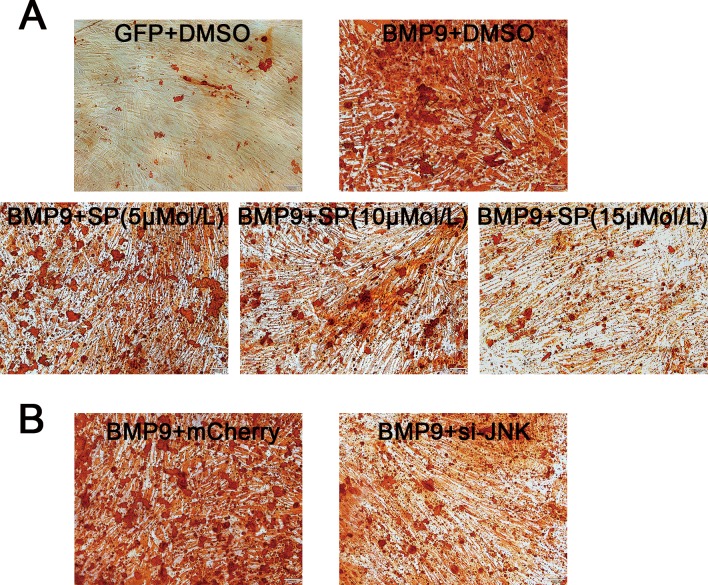
Inhibition of JNKs attenuates BMP9-induced extracellular mineralization of hPDLSCs. (**A**) Alizarin Red staining results showed that the JNK inhibitor SP600125 decreased the calcium deposition of cells stimulated by BMP9 in a dose-dependent manner three weeks post-osteo-induction. (**B**) Knock-down of JNKs by siRNA also reduced the formation of calcium nodules in hPDLSCs stimulated by BMP9.

Results of Alizarin Red S staining showed that treatment with the inhibitor SP600125 decreased the formation of calcific nodules in hPDLSCs stimulated by BMP9 in a dose-dependent manner after osteo-induction for three weeks (Fig 6A). Similarly, as illustrated in Fig 6B, JNKs knockdown had a negative effect on the BMP9-induced calcium deposition in hPDLSCs. Thus, JNKs possibly play a positive role in regulating BMP9-induced mineralization in hPDLSCs.

### Gene Silencing of JNKs Has a Negative Effect on BMP9-Induced Ectopic Bone Formation *In Vivo*

**Fig 7:**
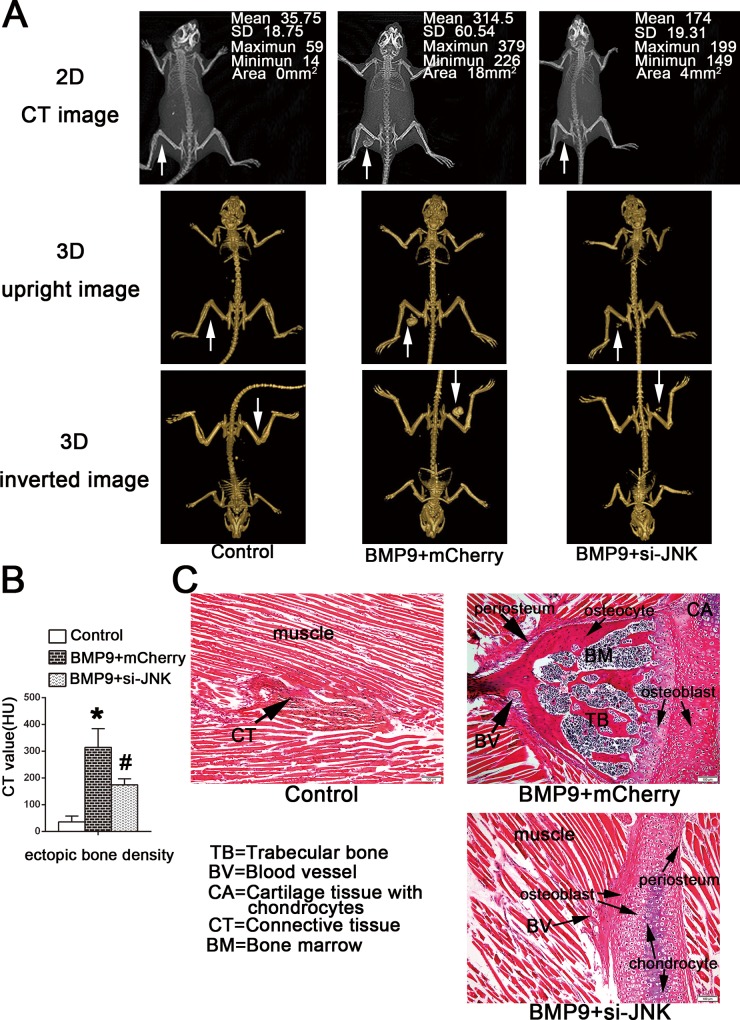
JNKs knockdown has a negative effect on BMP9-indcued heterotopic ossification in vivo. (**A**) Two months after implantation, the bone mineral density (BMD) of heterotopic ossification implants was analyzed by 3DVR software. Cross-section CT images and three-dimensional reconstruction images scanned by CBCT showed that si-JNK attenuated the bone mineral density (BMD) and volume of radiographic ectopic calcification in the musculature of mice compared to the BMP9 transfection group. (**B**) Quantitative analysis of BMD of ossification area in implanted region from 3D images reconstructed by an i-CAT system. n = 12; ‘‘*”, p<0.01 (vs. control group); ‘‘#”, p<0.01 (vs. BMP9 group). (**C**) H&E staining of the sections of the implants suggested that two months after transplantation, immature woven trabecular bone containing blood vessels, bone marrow tissues, osteocytes, and osteoblasts, were formed in the BMP9 transfection group, in which both endochondral ossification and subperiosteal ossification were involved in the formation of intramuscular ectopic bone. Gene silencing of JNKs inhibited osteogenic differentiation and mineralization of hPDLSCs stimulated by BMP9 *in vivo*. There were few new bone-like tissues consisting of bone matrix and osteoblasts, and more chondrocytes present in the si-JNK group compared to the BMP9 group.

hPDLSCs transduced by Ad-BMP9 or AdR-si-JNK were transplanted into the musculature of athymic mice. Two months after transplantation, as illustrated in (Fig 7A), two-dimensional CT and three-dimensional reconstructed radiography evaluation showed that BMP9 gene delivery to hPDLSCs induced the formation of a higher volume of bone-like tissues versus the GFP group *in vivo*. The quantitative measurement of BMD of implant sites suggested that JNKs knock down inhibited the maturation and mineralization of ectopic ossification induced by BMP9 overexpression, and these results are consistent with the in vitro results (Fig 7B; p<0.05). Furthermore, JNKs inhibition also reduced osteoid tissues mass stimulated by BMP9. Similarly, results of the histological examination were consistent with the radiography. As shown in (Fig 7C), treatment with BMP9 stimulated the formation of bone-like tissues, which consisted of immature woven trabecular bone containing osteogenic precursors, osteoblasts and bone matrix, while few mineralized nodules were detected in the GFP group. In addition, our histological results suggested that the ectopic ossification in the musculature originated from both endochondral ossification and subperiosteal ossification. Consistent with our radiography results, histological analysis also revealed that JNKs knock down inhibited BMP9-induced ossification mass, osteoblast maturation and osteoid matrix formation (Fig 7C). Few bone matrices and more chondrocytes were detected in the JNK gene silencing group.

### JNKs and Smad2/3 Pathway Crosstalk in BMP9-Mediated Osteogenesis

**Fig 8:**
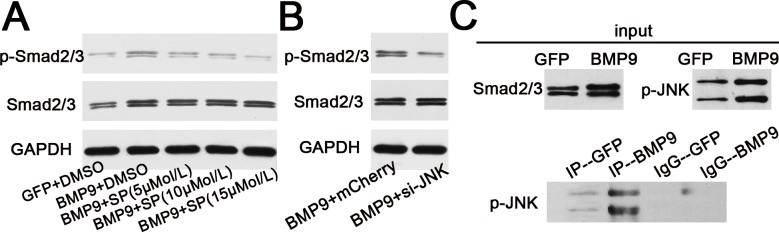
Crosstalk between JNKs and Smad2/3 signaling pathway in response to BMP9-induced osteogenic differentiation of hPDLSCs. (**A**) Western blotting results showed that treatment with the JNK inhibitor SP600125 at different concentrations (5, 10, or 15 mM) has a negative effect on BMP9-induced phosphorylation of Samd2/3, in a dose-dependent manner. (**B**) Western blotting results suggested that gene silencing of JNKs decreased the amount of p-Smadd2/3 in hPDLSCs stimulated by BMP9. (**C**) Western blotting results showed that BMP9 stimulated phosphorylation of JNKs and amount of Smad2/3 compared to the GFP group. The formation of protein complexes of Smad2/3 and p-JNK, detected by a co-IP assay, suggested the an interaction between Smad2/3 and p-JNK.

Our western blotting results detected a relationship between JNKs and the Smad2/3 pathway in BMP9-induced osteogenesis of hPDLSCs. First, we performed a western blotting assay to determine the activation of Smad2/3 stimulated by overexpression of BMP9, and the results showed that BMP9 stimulated the protein expression of p-Smad2/3 compared to the GFP group (Fig 8A). The protein expression of p-Smad2/3 stimulated by the overexpression of BMP9 was reduced by the addition of the JNKs inhibitor SP600125 in a dose-dependent manner (Fig 8A). Likewise, gene silencing of JNKs also had a negative effect on the expression of p- Smad2/3 in hPDLSCs according to western blot analysis (Fig 8B). Subsequently, as shown in (Fig 8C), a co-IP assay identified the formation of protein complexes of p-JNKs and Smad2/3 which suggested that Smad2/3 could directly interact with p-JNKs in hPDLSCs stimulated by BMP9. Taken together, these results tentatively confirm that there is crosstalk between JNKs and the Smad2/3 pathwy in BMP9-induced osteogenesis of hPDLSCs.

## Discussion

We investigated the effect of BMP9 on STRO-1^+^/CD146^+^ cells separated from PDL cell populations. To date, there is no specific surface marker that identifies PDLSCs. However, PDLSCs that are positive for STRO-1 (which recognizes an antigen on perivascular cells), and/or CD146 (which is expressed by endothelial cells), have greater colony-forming efficiency and osteogenic potential than STRO-1 and/or CD146 negative PDLSCs [13]. Our flow cytometry results showed that the isolation rate of STRO-1^+^/ CD146^+^ cells in PDLSCs was 27.9%, which was higher than the average percentage of STRO-1^+^/CD146^+^ in previous reports (2.4% or 2.6%) [14,15]. We think that differences in donor age, and healthy status, sites of tissues, and isolation methods may account for the difference in isolation rate of STRO-1^+^/CD146^+^ cells. Furthermore, the sorted STRO-1^+^/CD146^+^ cells in our study confirmed the potential of hPDLSCs to differentiate into osteoblast-like cells and adipocytes after osteogenic or adipogenic induction. Therefore, the isolated STRO-1^+^/CD146^+^ PDLSCs in our study exhibit multidifferentiation capacity and are ideal seed cells for periodontal regeneration.

BMP9, first isolated from fetal mouse liver, has been confirmed to be one of the most osteoinductive BMPs for induction of osteoblastic differentiation of MSCs in both in vitro and in vivo experiments utilizing adenovirus-transfection or recombinant human BMP9 methods [16–18]. As reported, gene delivery of BMP9 into osteoblast progenitors induced more rapid callus formation and a larger area of immature woven bone in mice models compared with BMP2, BMP6, and BMP7. In addition, BMP9-mediated osteogenesis resembled physiological bone healing during fracture repair [19]. We constructed recombinant adenovirus vectors to mediate BMP9 transfection into hPDLSCs and RT-PCR results showed that Ad-BMP9-transfected cells had stable BMP9 overexpression throughout the entire osteogenic differentiation process of hPDLSCs. Once activated, Runx2, a key transcription factor for osteogenesis, regulates the expression of early and late osteogenic-associated genes, such as ALP, OPN, BSP, and OCN [20,21]. ALP, another early marker of osteogenic differentiation, can hydrolyze phosphate—containing substrates to increase the local phosphate concentration, which is required for mineralization. We found that ALP activity was significantly increased by BMP9 overexpression at days 4, 7, and 10. To better understand the effect of BMP9 on osteogenic differentiation of hPDLSCs, we analyzed the expression levels of bone-specific genes using qRT-PCR. The early and late osteogenic mRNA levels of Runx2, ALP, OPN, and OCN were also up-regulated by BMP9 stimulation compared with the GFP control group at the indicated time points. BMP9 also induced the formation of more mineralized calcium nodules than were observed in the GFP control group after osteo-induction for three weeks. These findings strongly suggest that BMP9 promotes osteogenic differentiation and mineralization throughout the entire osteogenesis process of hPDLSCs.

Although much work has been performed to elucidate the signaling pathways of BMPs, the specific mechanisms underlying BMP9-mediated osteogenesis are poorly understood. As reported, BMP9 is resistant to noggin, a BMP antagonist, and BMP9 mediates osteoinduction through a mechanism that is different from that used by other BMPs [22,23]. Therefore, we are particularly interested in illuminating the signaling pathways involved in BMP9 osteoinductive activity. BMPs, as members of the TGF-β superfamily, bind to typeⅠand typeⅡ BMP receptors (BMPR-I or BMPR-II), and then primarily phosphorylate Smad1, Smad5 and Smad8, whereas both TGF-β and activin receptors activate Smad2 and Smad3 [24–26]. Indeed, the Smad signaling within a complex network of crosstalk is tightly integrated with other signaling pathways that largely contribute to modification of the initial Smad signals and allow the pleiotropic activities of BMPs. In addition to the Smad pathways, non-Smad pathways, including MAPK pathways, which are activated by BMPs, may play important roles in cell proliferation and differentiation. It has been confirmed that BMP9 most likely binds to BMPR-I proteins, such as ALK-1 and ALK-2, and phosphorylates the Smad pathway to regulate osteogenic gene expression in the nucleus [27,28]. JNKs, members of the MAPKs family, are also known as stress-activated protein kinase, and primarily induce apoptosis, but in some cellular systems, they also regulate proliferation and differentiation in response to extracellular stimulation [29]. JNKs play positive or negative roles in BMP2-induced osteogenesis [30–33]. Activation of JNKs is essential for the osteoinductive activity of BMP9 in MSCs [11]. We utilized the JNK inhibitor SP600125 as well as JNK1/2 knockdown by transfection with JNK1/2 siRNA into hPDLSCs and thereby investigated the role of JNK activation. We find, for the first time, that JNKs have a positive effect on the Runx2, ALP, OPN, and OCN gene expression induced by BMP9 in hPDLSCs. However, the precise cellular and subnuclear mechanism by which JNKs mediate the osteogenic differentiation potential of BMP9 in hPDLSCs is unknown. The MAPK pathways may engage in cross-talk with the Smad pathway and regulate the transcriptional levels of target genes [12,34]. JNK could phosphorylate Smad3 outside its -SSXS motif, further promoting Smad3 phosphorylation by the TGF-β receptor complex and its nuclear accumulation [12]. Similarly, TGF-β activates the JNK pathway, and thereafter directly phosphorylates smad2/3 at linker regions in the cytoplasm of normal stomach-origin cells [35]. In contrast, the TGF-β-activated ERK1/2 and JNK cascades negatively regulate Smad3-induced transcriptional activity as well as ALP activity and mineralization in osteoblasts [36]. We found that BMP9 induced the activation of JNK and Smad2/3 during osteogenic differentiation, and we noted cross-talk between JNKs and Smad2/3 during BMP9-induced osteogenic differentiation of hPDLSCs. Nevertheless, further details regarding the cross-talk of between JNKs and Smad2/3 and the downstream targeted nuclear transcription factors should be elucidated in the future.

Ex vivo or direct BMP9 gene therapy can promote endochondral bone formation in the paraspinal region [37,38]. Compared with direct gene delivery to the wound, stem-cell-based ex vivo gene delivery has some advantages, such as rapid cell proliferation, and sustained release of therapeutic factors, and it does not require direct injection of viral particles [39]. To further verify the immunogenicity and osteogenic potential of BMP9 in hPDLSCs in vivo, we demonstrate, for the first time, that hPDLSCs and BMP9 ex vivo gene therapy can promote the formation of ectopic bone-like tissues in the thigh musculature of immunodeficient nude mice. Because histological examination showed more chondrocytes in Ad-GFP injection sites and fewer residual chondrocytes after BMP9 transfection, we inferred that BMP9-stimulated ectopic bone formation progressed through endochondral bone formation. We also found that JNKs knock down inhibited BMP9-induced osteoblast differentiation and maturation in vivo.

In conclusion, we demonstrate the positive effect of the JNK pathway on BMP9-induced osteogenesis in hPDLSCs both in vitro and in vivo. JNKs likely engage in cross-talk with Smad2/3, regulating BMP9-induced osteogenesis. However, BMP9-stimulated osteogenesis involves many signaling pathways. To better regulate the ossification course, so that it is consistent with normal physiologic bone formation, the crosstalk between the primary Wnt pathway and MAPK pathways or the Notch pathway in relation to osteogenesis during BMP9-mediated osteogenesis still needs to be illuminated.

## Supporting Information

S1 Dataset Manuscript Data Set. (XLSX)
